# Novel Thiochroman‐4‐One Derivatives Bearing a Sulfonyl Hydrazone Moiety: Synthesis, Bioactivity, Molecular Simulation, and Drug‐Likeness Evaluation

**DOI:** 10.1002/cbdv.71884

**Published:** 2026-10-27

**Authors:** Pei Li, Wenming Pan, Tiantian Zhuang, Xiang Wang

**Affiliations:** ^1^ College of Life and Health Science Kaili University Kaili China; ^2^ Guizhou Key Laboratory of Miao Medicine, Qiandongnan Engineering and Technology Research Center for Comprehensive Utilization of National Medicine Kaili University Kaili China

**Keywords:** antioxidant and anti‐α‐glucosidase, drug‐likeness, molecular simulation, thiochroman‐4‐one

## Abstract

Herein, a series of novel thiochroman‐4‐one derivatives bearing sulfonyl hydrazone moiety were rationally designed and synthesized via molecular hybridization strategy. The sulfonyl hydrazone pharmacophore with excellent hydrogen‐bonding interaction ability was first introduced into the thiochroman‐4‐one parent skeleton to construct dual‐active functional molecules, aiming to achieve synergistic α‐glucosidase inhibition and antioxidant activity for antidiabetic therapy. All target compounds were structurally characterized by ^1^H NMR, ^1^ ^3^C NMR, and HRMS. Bioactivity results showed that multiple derivatives exhibited excellent α‐glucosidase inhibitory activity and antioxidant capacity, among which compound **5k** displayed the optimal comprehensive pharmacological performance with IC_50_ value of 4.57 µg/mL against α‐glucosidase, which was significantly superior to the positive control acarbose (237.48 µg/mL). Molecular docking and molecular dynamics (MD) simulation verified that the optimal compound **5k** could stably bind to the active pocket of α‐glucosidase through multiple hydrogen bonds and hydrophobic interactions, with lower binding energy and better complex stability. Drug‐likeness evaluation demonstrated that all synthesized derivatives complied with Lipinski's rule of five and had favorable pharmacokinetic properties. This work provides a novel structural scaffold for the development of high‐efficiency antioxidant, α‐glucosidase, and antibacterial inhibitors, and demonstrates its potential as a lead compound for pharmaceutical and agrochemical development.

## Graphical abstract

Compound **5k** demonstrated outstanding in vitro anti‐α‐glucosidase activity, with an IC_50_ value of 4.57 µg/mL, which was approximately 51‐fold lower than that of acarbose (237.48 µg/mL). Molecular docking and MD simulations indicated that compound **5k** forms a stable interaction with human intestinal α‐glucosidase.

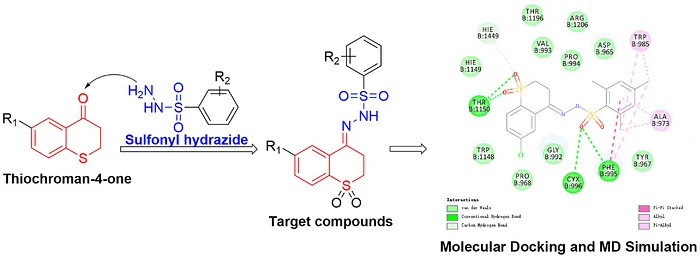

## 1 Introduction

Free radicals and reactive oxygen species (ROS) participate in diverse physiological processes [1]. Under homeostatic conditions, endogenous antioxidant systems maintain a balanced redox state to protect lipids, proteins, and DNA from oxidative injury [2]. However, endogenous and exogenous stressors—including aging, chronic inflammation, and unhealthy lifestyles—can disrupt this equilibrium, leading to excessive ROS accumulation and oxidative damage [3]. Such oxidative stress is closely associated with aging, diabetes, neurodegenerative disorders, and tumorigenesis [4], driving continuous demand for potent and safe antioxidant agents.

Type 2 diabetes mellitus (T2DM) is a global metabolic disorder characterized by insulin deficiency and insulin resistance [5]. α‑Glucosidase inhibitors (e.g., acarbose, miglitol, voglibose) are first‑line oral drugs that delay carbohydrate hydrolysis in the small intestine, thereby suppressing postprandial hyperglycemia [6]. Nevertheless, their clinical use is often limited by gastrointestinal side effects such as bloating, diarrhea, and abdominal discomfort [7]. Therefore, the discovery of novel α‑glucosidase inhibitors with enhanced efficacy and reduced toxicity remains a priority in antidiabetic drug development.

As a classic sulfur‐containing heterocyclic compound, thiochroman‐4‐one has unique rigid spatial structure and good biological compatibility, and its derivatives have been proven to possess antidiabetic, antioxidant, antibacterial, and anti‐inflammatory activities in previous studies [8, 9, 10]. Numerous studies have reported that thiochroman‐4‐one derivatives modified with hydroxyl, carboxyl, and heterocyclic groups can exert moderate α‐glucosidase inhibitory activity [11, 12, 13, 14, 15]. However, the existing modified structures still have obvious shortcomings: most traditional derivatives only rely on the single skeleton effect to exert enzymatic inhibition, lack of auxiliary pharmacophores for hydrogen bond recognition, resulting in weak binding affinity with α‐glucosidase; in addition, few studies have combined the antioxidant stress function of thiochroman‐4‐one with antidiabetic effects, ignoring the synergistic therapeutic potential for diabetic oxidative damage complications. Moreover, the substituent selection of existing analogs is random, without systematic structural design and structure–function relationship (SAR) verification, leading to unclear pharmacological mechanism and low druggability. In 2026, we reported a series of novel thiochroman‐4‐one derivatives containing a semicarbazone moiety as potential α‐glucosidase inhibitors (Figure 1) [16]. Separately, sulfonyl hydrazone motifs have emerged as privileged pharmacophores in medicinal chemistry, frequently serving as central frameworks in drug discovery efforts [17]. Owing to their versatile bioactivity profile, including radical‐scavenging capacity, antimicrobial potency, antiproliferative action, and α‐glucosidase inhibition, sulfonyl hydrazone derivatives have attracted increasing attention in both academic and industrial research settings over the past several years [18, 19, 20, 21].

**FIGURE 1:**
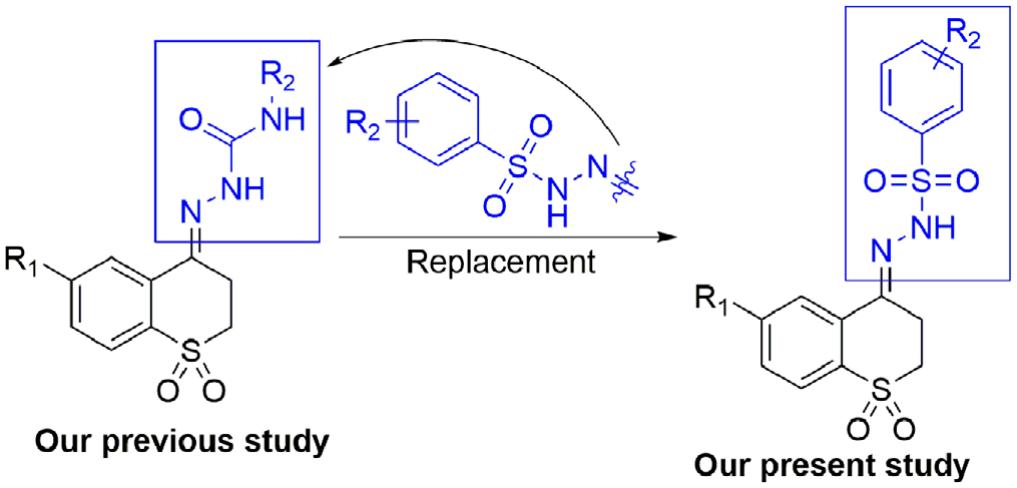
Rational design of the target compounds.

To solve the above problems, this study innovatively introduces sulfonyl hydrazone moiety into the thiochroman‐4‐one parent skeleton for the first time based on molecular hybridization principle (Figure 1). The sulfonyl hydrazone group contains multiple N─H and S═O polar functional groups, which can form abundant hydrogen bond and electrostatic interactions with amino acid residues in the active pocket of α‐glucosidase, effectively improving the target binding ability of parent skeleton. Meanwhile, the sulfonyl hydrazone fragment has inherent excellent antioxidant activity, which can cooperate with the antidiabetic effect of thiochroman‐4‐one to alleviate oxidative stress injury in diabetic patients, realizing dual‐target synergistic therapy. Different from the single‐function thiochroman‐4‐one derivatives reported in previous literature, the newly constructed dual‐functional structural framework not only optimizes the enzymatic inhibitory activity, but also compensates for the deficiency of single pharmacological effect of traditional analogs, which is the core innovation of this study. Furthermore, we designed and screened different R_1_ and R_2_ substituent combinations based on pharmacophore characteristics, systematically explored the influence of substituent electronic effect and steric hindrance on biological activity, and finally obtained high‐efficiency lead compounds with good drug‐likeness.

## 2 Results

### 2.1 Chemistry and Spectral Characterization

As shown in Scheme 1, the target compounds **5a–5k** were prepared via a five‑step synthetic route starting from 4‑substituted thiophenols, involving substitution, intramolecular cyclization, oxidation, hydrazidation, and condensation (Scheme 1). Overall yields ranged from 60% to 81%. Structures were fully characterized by ^1^H NMR, ^1^ ^3^C NMR, and HRMS. Taking compound **5k** as an example: in the ^1^H NMR spectrum, two characteristic singlets at *δ* 9.76 and 6.74 ppm were assigned to the N─H and aromatic protons of the sulfonyl hydrazone group. The ^1^ ^3^C NMR signals at *δ* 155.22 and 143.73 ppm corresponded to C═O and C═N carbons, respectively. HRMS detected an [M+Na]^+^ peak at *m*/*z* 332.10326, consistent with the calculated molecular mass.

**SCHEME 1:**
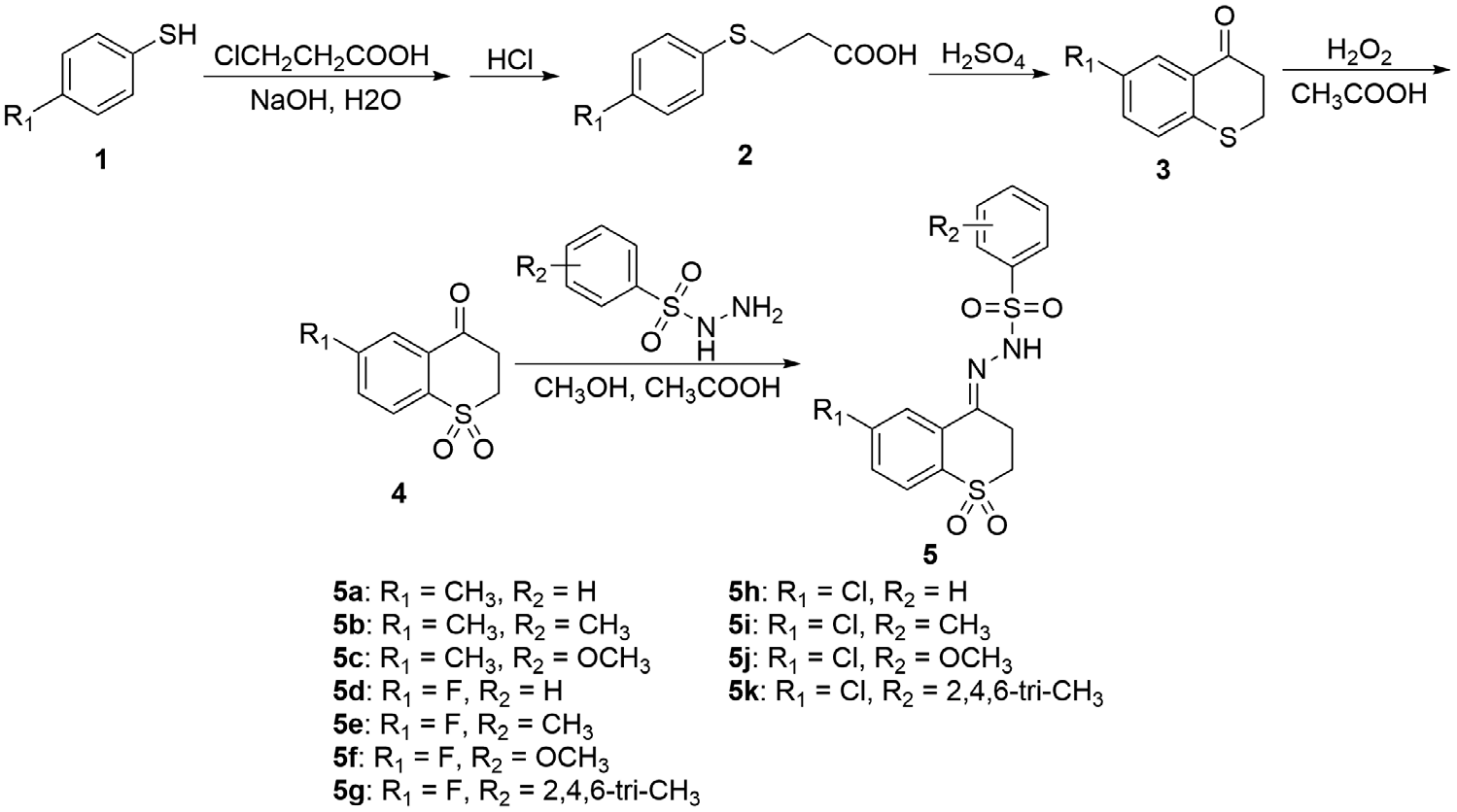
The synthetic route of the target compounds **5a**−**5k**.

### 2.2 Antioxidant and Anti‐α‐Glucosidase Test

As presented in Table 1, the synthesized compounds **5a–5k** exhibited diverse levels of in vitro antioxidant activity, with DPPH radical scavenging rates ranging from 2.11% to 18.71% at 40 µg/mL, and ABTS radical scavenging rates ranging from 10.98% to 43.13% at 50 µg/mL, both of which were lower than those of the reference standards vitamin C and trolox.

**TABLE 1:** In vitro antioxidant activities of the target compounds **5a–5k**.

Compounds	Scavenging activity (%)^a^	
	DPPH	ABTS
**5a**	4.07 ± 1.11	16.60 ± 1.26
**5b**	2.11 ± 1.02	10.98 ± 0.75
**5c**	8.11 ± 1.25	23.37 ± 1.29
**5d**	11.36 ± 2.04	22.27 ± 1.78
**5e**	6.92 ± 1.24	15.53 ± 1.55
**5f**	15.80 ± 1.20	36.71 ± 2.02
**5g**	4.21 ± 0.29	11.26 ± 1.17
**5h**	15.54 ± 0.86	28.01 ± 1.23
**5i**	12.46 ± 1.01	18.92 ± 1.65
**5j**	18.71 ± 0.98	43.13 ± 2.22
**5k**	8.55 ± 0.75	13.13 ± 2.86
Vitamin C	90.20 ± 2.25	/
Trolox	/	100

^a^ “/” means no determined.

Table 2 reveals that the synthesized compounds **5a–5k** exhibited moderate to strong in vitro inhibitory effects against α‐glucosidase at 50 µg/mL, with inhibition rates ranging from 32.47% to 95.57%. Notably, compounds **5b**, **5g**, **5i**, and **5k** demonstrated excellent in vitro inhibitory potency against α‐glucosidase, with IC_50_ values of 26.76, 10.26, 37.81, and 4.57 µg/mL, respectively, which were lower than that of the reference drug acarbose (237.48 µg/mL). Among them, compound **5k** showed the best in vitro inhibitory effect against α‐glucosidase, with an IC_50_ value of 4.57 µg/mL, which was nearly 51 times lower than that of the reference drug acarbose.

**TABLE 2:** In vitro anti‐α‐glucosidase activities of the target compounds **5a–5k**.

Compounds	Anti‐α‐glucosidase activity (%)	IC_50_ (µg/mL)^a^
**5a**	32.47 ± 1.57	/
**5b**	89.17 ± 1.94	26.76 ± 1.65
**5c**	42.53 ± 1.68	/
**5d**	25.96 ± 1.05	/
**5e**	39.14 ± 2.19	/
**5f**	36.61 ± 1.07	/
**5g**	92.54 ± 1.04	10.26 ± 2.24
**5h**	38.46 ± 1.55	/
**5i**	82.42 ± 2.05	37.81 ± 2.14
**5j**	47.01 ± 2.08	/
**5k**	95.57 ± 1.11	4.57 ± 0.58
Acarbose	53.00 ± 1.65	237.48 ± 1.56

^a^ “/” means no determined.

### 2.3 Molecular Docking

Molecular docking was performed to further investigate the binding modes of compound **5k** and human intestinal α‐glucosidase (PDB ID: 3TOP) using AutoDock Tools 1.5.6 with the protocol described in our previous study [22]. As depicted in Figure 2, molecular docking analysis indicates that compound **5k** demonstrates a binding energy of −6.8 kcal/mol during its interaction with human intestinal α‐glucosidase (PDB ID: 3TOP). Inside the 3TOP protein, compound **5k** predominantly interacts with the amino acid residues HIE1149, THR1196, VAL993, ARG1206, PRO994, ASP965, TYR967, GLY992, PRO968, and TRP1148 via van der Waals forces. THR1150, CYX996, and PHE995 form conventional hydrogen bonds, while HIE1449 creates a carbon–hydrogen bond with compound **5k**. A π–π stacked interaction is detected between PHE995 and compound **5k**, while TRP985 and ALA973 participate in alkyl and π–alkyl interactions with compound **5k**, respectively.

**FIGURE 2:**
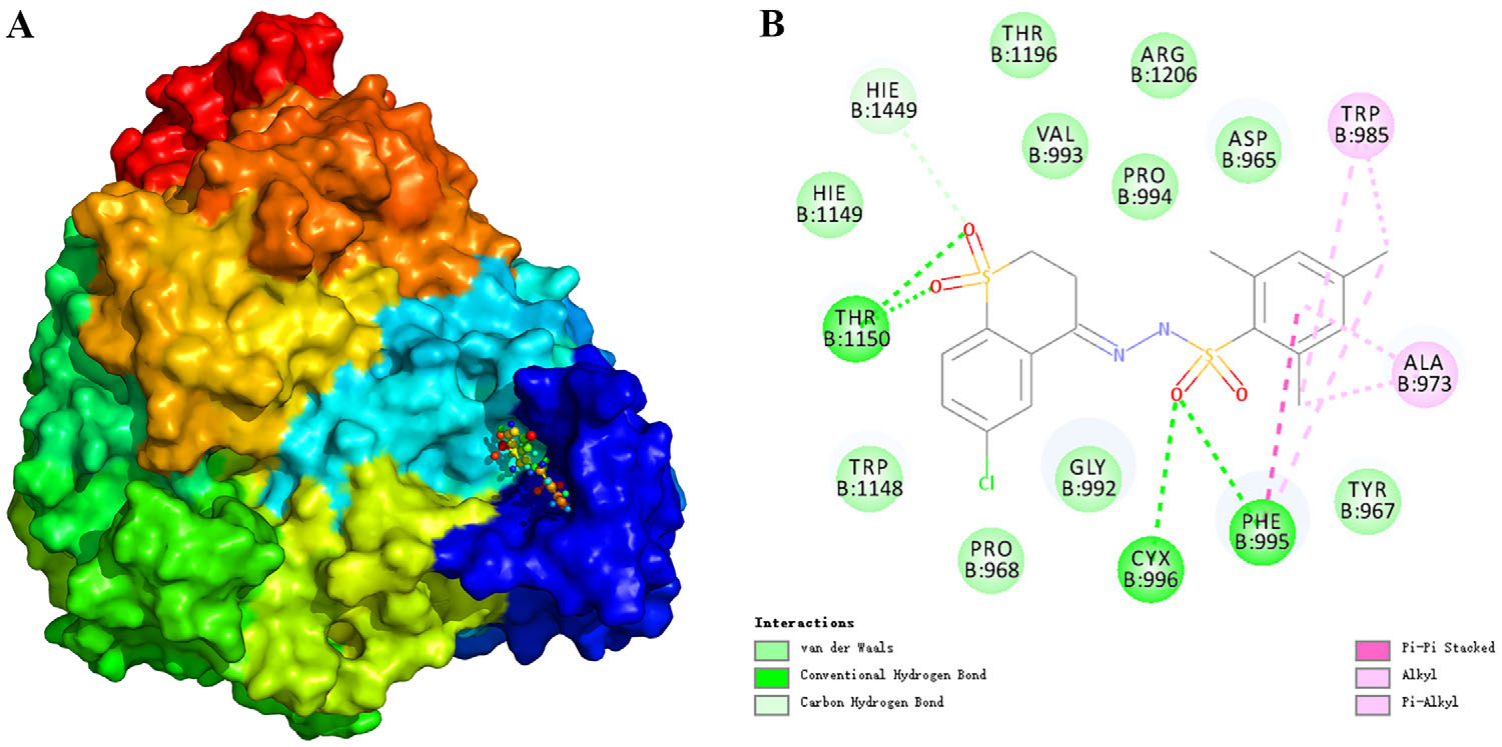
The 3D (A) and 2D (B) molecular binding mode between compound **5k** and the active site of human intestinal α‐glucosidase.

### 2.4 Molecular Dynamics (MD) Simulation

In recent years, the significance of MD simulations has grown substantially in the fields of molecular biology and drug development [23]. These computational methods, which offer atomic‐level resolution and high temporal precision, allow for detailed observation of the dynamic behavior of receptors and their binding ligands [24]. The advancements in simulation efficiency, accuracy, and user‐friendliness, along with the increasing availability of experimentally determined structures, have rendered active molecular modeling more appealing to experimental researchers. MD simulations have become a vital tool for clarifying the functional mechanisms of active molecules, identifying the structural underpinnings of diseases, and promoting the design and optimization of small‐molecule drugs, peptides, and engineered proteins [25]. In this study, MD simulations of protein–ligand complexes were conducted using GROMACS 2022.2 to explore the molecular interactions between the receptors and their binding ligands [26, 27].

Root mean square deviation (RMSD) is employed to measure the difference between the receptors and ligands. During the simulation process, it is used to track the changes of the molecular structure relative to the initial structure and to observe whether the range of change tends to stabilize [28]. A lower RMSD value indicates a higher degree of similarity between the receptors and ligands [29]. As shown in Figure 3A, the RMSD of the complex structure gradually stabilizes as the simulation progresses, indicating that the complex structure is gradually achieving stability.

**FIGURE 3:**
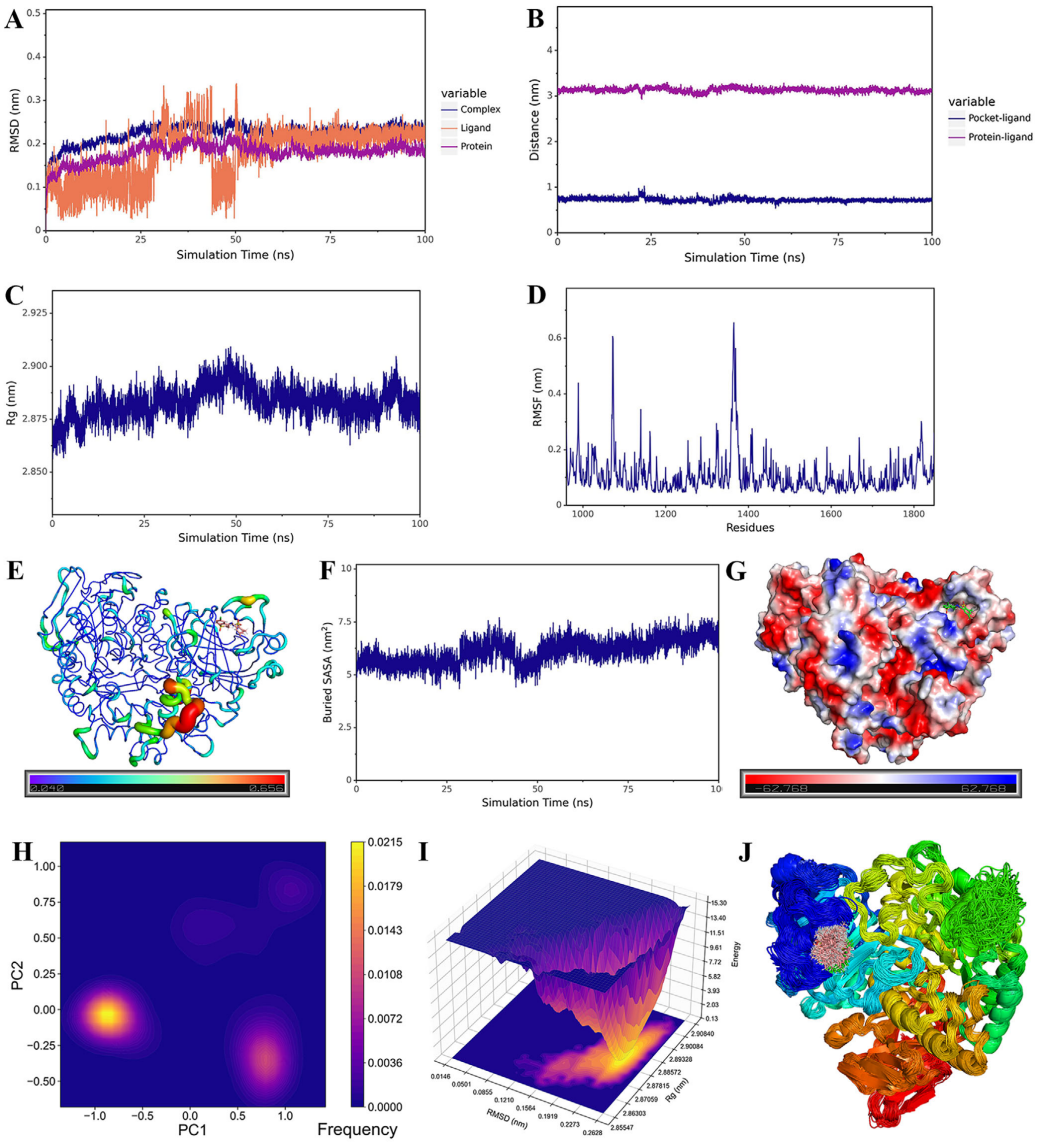
The RMSD (A), distance (B), *R*_g_ (C), RMSF (D), B‐factor (E), buried SASA (F), surface electrostatic potential (G), PCA (H), FEL (I), and simulated conformational superposition (J) of compound **5k** binding to the active site of human intestinal α‐glucosidase.

By analyzing the distance between the centroid of the residues at the initial docking sites and the centroid of the ligands, the binding state of the ligands to the receptors can be determined [30]. As shown in Figure 3B, both the distance between the small molecule and the center of the protein and the distance between the ligand and the binding site of receptor gradually reach a steady state. This suggests that the ligand binds stably to the binding site of receptor, and the binding of the ligand to the receptor gradually stabilizes.

The radius of gyration (*R*_g_), defined as the root mean square distance of all atoms in a molecule from its center of mass, reflects the distribution range of atoms within the molecule relative to the center of mass [31]. It serves as a crucial parameter for assessing the compactness of the overall structure of receptor‐ligand complexes [32]. A smaller *R*_g_ value implies a more compact molecular structure, whereas a larger *R*_g_ value suggests a looser structure, potentially in a fluffy state [33]. As depicted in Figure 3C, the *R*_g_ of the receptor–ligand complex gradually reaches a stable state as the simulation advances, indicating that the structure of the complex is gradually stabilizing.

Root mean square fluctuation (RMSF) is employed to compute the fluctuations of each atom with respect to its average position. It characterizes the average of structural alterations over time and offers a depiction of the flexibility of diverse regions of the receptor [33]. As can be clearly discerned from Figure 3D, the average RMSF values of the proteins in the complex group are 0.040 nm, with the maximum peak attaining 0.656 nm. This suggests that the binding between the receptor and ligand is stable. Meanwhile, taking the RMSF value as the B‐factor, a B‐factor map was constructed. The result, as shown in Figure 3E, indicates that the flexibility of the amino acids around the small molecule is relatively low, thus the binding between the receptor and ligand is relatively stable.

Buried solvent accessible surface area (buried SASA) is a metric used to assess which parts of a receptor–ligand complex are buried internally and inaccessible to solvents [34]. A larger value of buried SASA indicates stronger interactions and a larger contact area between the receptor and ligand [35]. As shown in Figure 3F, buried SASA gradually stabilizes, suggesting that the contact area between the receptor and ligand gradually stabilizes.

Surface electrostatic potential reflects the charge properties (such as positive, negative, or neutral) of different regions on the receptor surface, thereby influencing the interaction between the receptor and the ligand [33]. It is an important physical quantity for describing the charge distribution on the molecular surface and its impact on the external environment [36]. As shown in Figure 3G, the ligand binds to the receptor groove, and the surface of the binding site mainly exhibits electrical neutrality, positive charge, and negative charge, which facilitates the formation of electrostatic interactions and hydrogen bonds.

Principal component analysis (PCA), a method for reducing data dimensions and extracting major motion patterns, simplifies complex high‐dimensional molecular motion data into a few principal components (PCs) and captures the most significant motion patterns of molecules during simulations [37]. The PCA analysis of the trajectories of the ligand within the receptor–ligand complex produces the results presented in Figure 3H. In the PCA plot, two distinct clusters are identified, which correspond to the conformations of the ligand exhibiting higher occurrence frequencies. Moreover, the distribution of these conformations is relatively concentrated, indicating that the conformation of the ligand remains relatively stable. It is noteworthy that a red cluster with the highest occurrence frequency is discernible in the PCA plot, which represents the conformation of the ligand in a relatively stable binding state with the receptor.

The free energy landscape (FEL) in MD simulation serves as a graphical depiction of the free energy distribution of a molecular system across different conformational states. This representation can elucidate the diverse possible conformations of a molecule or system, along with their relative stabilities [38]. By using the RMSD and *R*_g_ of the receptor–ligand complex to create a free energy topography of the complex, the results are shown in Figure 3I. The results indicate that there is one low‐energy state in the receptor–ligand complex, suggesting that the overall structure of the complex is relatively stable.

The simulated trajectories were processed and the simulated conformations were superimposed. As shown in Figure 3J, the results indicated that the superimposition degree of the receptor–ligand complex was relatively high, suggesting that the ligand remained bound to the receptor throughout.

Hydrogen bond, an important interaction force for the binding of receptor and ligand, is related to electrostatic interactions and can reflect the strength of electrostatic interactions [39]. As shown in Figure 4A, the number of hydrogen bonds between the receptor and ligand fluctuates basically between 0 and 3. Meanwhile, hydrogen bond frequency analysis is a common method for studying the formation and maintenance of hydrogen bonds between the receptor and ligand [40]. Through the analysis of hydrogen bond frequency, it is evident that a ligand forms a high‐frequency hydrogen bond with a specific amino acid residue at the binding site, which suggests the crucial role of this hydrogen bond in the binding process. As shown in Table 3 and Figure 4B, there are stable hydrogen bond pairs between the receptor and the ligand, such as PHE995:Ligand, with an existence frequency of 50.6% and strong stability.

**FIGURE 4:**
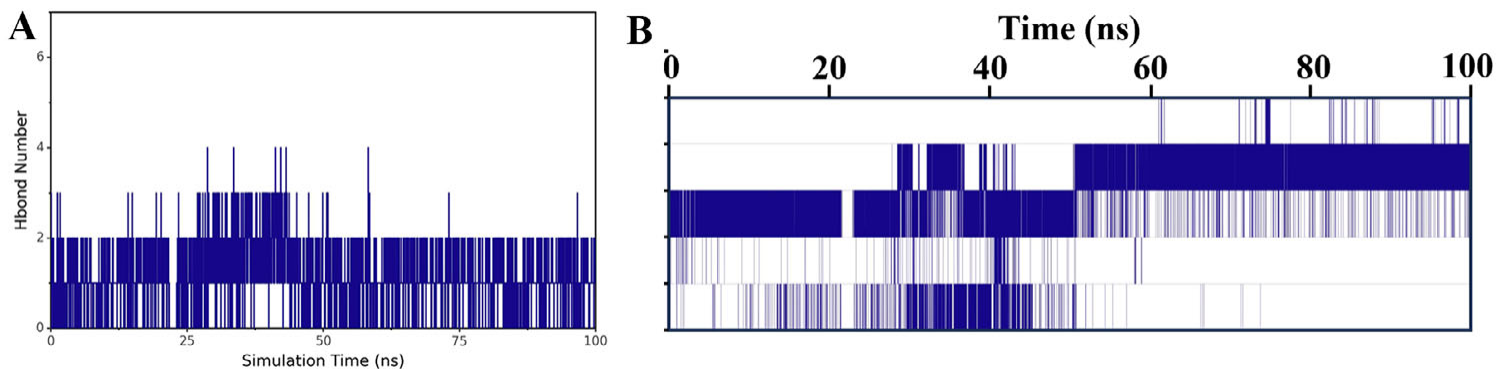
Hydrogen bond numbers (A) and of the formation frequency of hydrogen bond (B) of compound **5k** binding to the active site of human intestinal α‐glucosidase.

**TABLE 3:** The occupancy rates of the hydrogen bond pair between compound **5k** and human intestinal α‐glucosidase.

Hydrogen bond donor	Hydrogen bond acceptor	Occupancy rate (%)
ASN989 (chain A#ND2)	Ligand (14025#O)	1.3
PHE995 (chain A#N)	Ligand (14024#O)	50.6
PHE995 (chain A#N)	Ligand (14025#O)	44.8
Ligand (14003#N)	965ASP (chain A#OD1)	2.4
Ligand (14003#N)	965ASP (chain A#OD2)	13.6

Without considering solvation, the van der Waals forces and electrostatic interaction forces between the receptor and ligand in the complex were calculated, and the changes in the binding forces during the simulation process were also analyzed. As shown in Figure 5A, van der Waals forces and hydrophobic interactions (VDW) and electrostatic interactions (ELE) in the complex gradually stabilize as the simulation proceeds, indicating that the binding between the receptor and ligand gradually stabilizes. Meanwhile, the binding energy was decomposed to obtain the contribution of each amino acid to the overall binding energy, and to evaluate the important amino acids in the receptor. The residues in the receptor that contribute significantly to their respective binding energies are shown in Figure 5B. From this figure, it is evident that the key amino acids involved in ligand binding within the receptor encompass PRO994, THR1150, and so forth.

**FIGURE 5:**
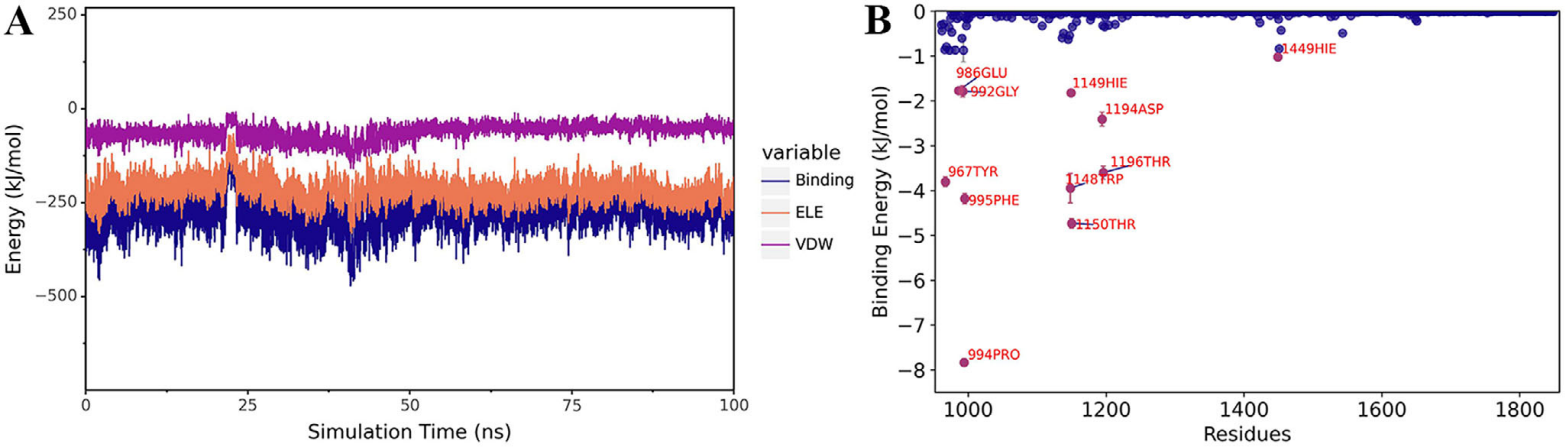
The FEL free energy (A) and analysis of residue contribution (B) of compound **5k** binding to the active site of human intestinal α‐glucosidase.

Considering the solvation energy, the complex trajectories in the stable state were selected by comprehensively evaluating RMSD, *R*_g_, distance, buried SASA, and interaction energy. The molecular mechanics poisson−boltzmann surface area (MMPBSA) method was used to calculate the energy terms related to the binding energy between the receptor and ligand [41]. As presented in Table 4, it is evident that the van der Waals interaction energy (Δ*E*_vdw_) in the receptor–ligand complex is greater than the electrostatic energy (Δ*E*_ele_), and the Δ*E*_ele_ is comparable to the hydrophobic interaction energy (Δ*E*_nonpol_). Therefore, in the composition of the binding energy, the van der Waals interaction plays a dominant role, whereas the electrostatic interaction and hydrophobic interaction play a subordinate role. The Δ*E*_MMPBSA_ of the receptor and the ligand is −55.975 kJ/mol, suggesting a relatively high binding energy and affinity between the receptor and the ligand.

**TABLE 4:** The occupancy rates of the hydrogen bond pair between compound **5k** and human intestinal α‐glucosidase.

Independent components	Energy (KJ/mol)
Van der Waals energy (Δ*E*_vdw_)	−151.246 ± 1.841
Electrostatic energy (Δ*E*_ele_)	−17.847 ± 1.067
Polar solvation energy (Δ*E*_pol_)	131.811 ± 2.662
Nonpolar solvation energy (Δ*E*_nonpol_)	−18.693 ± 0.201
Total binding energy (Δ*E*_MMPBSA_)	−55.975 ± 0.565
−*T*∆*S*	6.215 ± 2.009
Total binding free energy (Δ*G*_bind_)	−49.76 ± 2.552

### 2.5 ADMET and Drug‐Likeness Profiles

As shown in Table S1, ADMET prediction via Deep‑PK suggested that compound **5k** has favorable absorption, distribution, metabolism, excretion, and safety profiles. In parallel, its drug‐likeness was assessed using Lipinski's rule of five—a widely accepted guideline for estimating oral bioavailability. According to this rule, a molecule is more likely to be orally bioavailable if it meets at least three of the following four criteria: molecular weight ≤ 500 Da, calculated log*P* ≤ 5, no more than 5 hydrogen bond donors, and no more than 10 hydrogen bond acceptors [42]. As detailed in Table S2, drug‑likeness assessment using Lipinski's rule of five showed that compound **5k** fully satisfies all four criteria, supporting good oral bioavailability potential. These results provide a scientifically grounded basis for subsequent structural refinements—focused on improving pharmacokinetic behavior without compromising its favorable safety profile.

### 2.6 Antibacterial Activity Test

In addition to the core antidiabetic pharmacological evaluation (α‐glucosidase inhibition and antioxidant activity), this study additionally evaluated the auxiliary antibacterial activity of the target compounds against *Xanthomonas axonopodis* pv. *citri* (*Xac*). As presented in Table 5, compounds **5a–5k** exhibited good in vitro antibacterial activity against *Xac*, with inbihiton rates ranging from 65.22% to 95.65% at 200 µg/mL and from 42.29% to 80.26% at 100 µg/mL, respectively, both of which were higher than those of bismerthiazol and thiodiazole copper. Among them, compound **5j** showed the best in vitro antibacterial activity against *Xac* with the EC_50_ value of 15.67 µg/mL, which was lower those of bismerthiazol (143.65 µg/mL) and thiodiazole copper (226.32 µg/mL).

**TABLE 5:** In vitro antibacterial activity against *Xac* of the target compounds **5a–5k**.

Compounds	Inhibition rate (%)		EC_50_ (µg/mL)
	200 µg/mL	100 µg/mL	
**5a**	77.56 ± 1.65	56.54 ± 1.27	52.65 ± 3.32
**5b**	65.29 ± 0.95	42.29 ± 0.58	66.65 ± 3.31
**5c**	80.15 ± 2.05	60.15 ± 2.27	34.65 ± 2.26
**5d**	85.69 ± 1.10	64.36 ± 1.95	28.69 ± 2.29
**5e**	78.26 ± 2.29	56.64 ± 1.65	53.26 ± 2.74
**5f**	89.65 ± 1.65	70.26 ± 1.25	25.65 ± 3.05
**5g**	65.22 ± 1.25	43.26 ± 2.95	67.02 ± 2.29
**5h**	92.26 ± 1.59	75.69 ± 3.18	20.26 ± 2.25
**5i**	87.59 ± 1.01	66.56 ± 1.59	28.36 ± 1.95
**5j**	95.65 ± 1.58	80.26 ± 1.88	15.67 ± 2.22
**5k**	70.29 ± 2.24	48.95 ± 2.65	60.35 ± 2.58
Bismerthiazol	56.69 ± 1.65	32.95 ± 2.69	143.65 ± 2.29
Thiodiazole copper	37.59 ± 2.21	22.68 ± 0.23	226.32 ± 1.95

## 3 Discussion

### 3.1 Rational Design of the Target Compounds

In this study, a series of novel dual‐functional thiochroman‐4‐one derivatives bearing sulfonyl hydrazone moiety were successfully designed and synthesized via a molecular hybridization strategy, which effectively addressed the key defects of traditional thiochroman‐4‐one analogs including single pharmacological function, unclear structure optimization logic, and weak target binding affinity. Different from the random substituent modification adopted in most previous studies, the molecular design in this work is completely based on the pharmacophore characteristics of α‐glucosidase and the pathological mechanism of diabetic oxidative stress, with definite targeted optimization objectives. The active pocket of human intestinal α‐glucosidase is composed of hydrophobic amino acid residues and acidic polar residues, which requires inhibitor molecules to possess both rigid hydrophobic skeleton and abundant hydrogen‐bonding donor–acceptor sites to achieve stable binding and enzymatic inhibition. The thiochroman‐4‐one parent skeleton selected in this study provides excellent hydrophobic matching ability for the hydrophobic region of the α‐glucosidase active pocket, while the introduced sulfonyl hydrazone fragment contains rich N─H and S═O polar functional groups, which can form hydrogen bonds and electrostatic interactions with the target pocket. The superposition of the two pharmacophores realizes the organic combination of hydrophobic recognition and polar interaction, fundamentally improving the target affinity of the parent skeleton.

The selection of R_1_ and R_2_ substituents follows the dual optimization principle of enzymatic inhibitory activity and antioxidant performance. For the R_2_ substituent on the thiochroman‐4‐one skeleton, short‐chain alkyl and halogen groups were preferentially screened due to their low steric hindrance and high electronegativity, which can maintain the structural flexibility of the skeleton and enhance molecular polarization, thereby optimizing the binding effect with α‐glucosidase. Excessively large substituents were abandoned because their steric hindrance would block the entry of molecules into the enzyme active pocket and reduce biological activity. For the R_1_ substituent on the sulfonyl hydrazone benzene ring, electron‐withdrawing and electron‐donating groups were designed comparatively to regulate the dual pharmacological activities of the compounds. Electron‐withdrawing substituents can enhance the hydrogen‐bonding ability of sulfonyl hydrazone groups to improve enzymatic inhibition, while moderate electron‐donating groups optimize free radical scavenging capacity, realizing the complementary balance of hypoglycemic and antioxidant activities. This targeted substituent design strategy provides a clear theoretical basis for the significant activity differences of the synthesized derivatives.

### 3.2 In Vitro Bioactivity Analysis

In vitro bioassay results verified the successful construction of dual‐active hypoglycemic and antioxidant molecules in this work. All synthesized compounds exhibited certain in vitro antioxidant and α‐glucosidase inhibitory activities, and several derivatives showed excellent pharmacological performance far exceeding the positive control drug acarbose. Notably, compound **5k** exhibited the optimal α‐glucosidase inhibitory activity with an IC_50_ value of 4.57 µg/mL, which was approximately 17‐fold more potent than the recently reported thiochroman‐4‐one derivative ((*E*)‐*N*‐isopropyl‐2‐(6‐methyl‐1,1‐dioxidothiochroman‐4‐ylidene)hydrazine‐1‐carboxamide), which exhibited an IC_50_ of 84.65 µg/mL against α‐glucosidase [16]. The ultra‐high inhibitory activity of compound **5k** fully confirms the rationality of sulfonyl hydrazone pharmacophore modification, proving that the hybrid structure can effectively break through the activity bottleneck of traditional thiochroman‐4‐one derivatives.

Notably, the antioxidant activity of the target compounds was generally lower than that of vitamin C and trolox, which is consistent with the core design orientation of this study. Different from specialized antioxidant drugs, the compounds in this work take hypoglycemic activity as the core function, and antioxidant activity serves as an auxiliary therapeutic mechanism for diabetic complications. Importantly, α‐glucosidase inhibition and antioxidant activity exert a synergistic hypoglycemic mechanism in the treatment of type 2 diabetes, which jointly improves the disorder of glucose metabolism and oxidative microenvironment in diabetic patients. Mechanistically, intestinal α‐glucosidase is a key rate‐limiting enzyme for carbohydrate hydrolysis; its overexpression and hyperactivity can accelerate the decomposition of dietary starch and disaccharides into glucose, leading to rapid postprandial blood glucose spikes, which is the direct cause of hyperglycemia. The synthesized sulfonyl hydrazone‐modified thiochroman‐4‐one derivatives can competitively bind to the active pocket of α‐glucosidase, block the catalytic hydrolysis pathway of carbohydrates, and fundamentally delay intestinal glucose absorption to achieve a direct hypoglycemic effect.

On the other hand, long‐term persistent hyperglycemia can trigger excessive accumulation of intracellular ROS, resulting in severe oxidative stress. Excess ROS will oxidatively damage pancreatic β‐cells, reduce insulin synthesis and secretion, and simultaneously induce insulin resistance in peripheral tissues such as liver and muscle, further exacerbating glucose metabolic disorders and forming a vicious cycle of “hyperglycemia‐oxidative stress‐insulin resistance”. However, in terms of antioxidant activity, most of the traditional thiochroman‐4‐one derivatives only have weak DPPH and ABTS radical scavenging ability. The derivatives synthesized in this study retain the excellent antioxidant characteristics of sulfonyl hydrazone moiety, and most compounds show medium to strong antioxidant activity, which is consistent with our initial design expectation of “dual antioxidant and hypoglycemic synergistic effect”.

In addition, this study innovatively evaluated the auxiliary antibacterial activity of the target compounds against *Xac*. The results showed that most derivatives had excellent antibacterial activity, and compound **5j** displayed the best antibacterial effect with an EC_50_ of 15.67 µg/mL, which was significantly superior to commercial bactericides bismerthiazol and thiodiazole copper. It is necessary to clarify that the antibacterial activity is an independent and complementary pharmacological property, which is not involved in the antidiabetic mechanism and does not interfere with the SAR of hypoglycemic activity. This multi‐functional characteristic endows the novel thiochroman‐4‐one derivatives with dual application prospects in pharmaceutical antidiabetic and agricultural bactericidal fields, expanding the application scope of such heterocyclic compounds.

### 3.3 SAR Analysis

Based on the differential bioactivity data of 11 target derivatives, a complete and systematic structure–activity relationship was summarized to clarify the intrinsic correlation between structural modification and pharmacological activity, providing precise guidance for subsequent structural optimization of lead compounds. First, for the R2 substituent of the thiochroman‐4‐one parent skeleton, fluorine‐substituted derivatives showed significantly better comprehensive activity than methyl‐substituted analogs. The strong electronegativity of fluorine atoms can optimize the molecular charge distribution, enhance the electrostatic interaction between the molecule and the α‐glucosidase active pocket, and improve the stability of ligand–protein binding. In contrast, unsubstituted or large‐volume substituted thiochroman‐4‐one derivatives have increased molecular flexibility and steric hindrance, resulting in decreased target binding ability and weakened enzymatic inhibitory activity.

Second, the electronic effect of R_1_ substituents on the sulfonyl hydrazone benzene ring plays a decisive role in regulating dual activities. Electron‐withdrawing modified derivatives exhibited prominent α‐glucosidase inhibitory activity, which is attributed to the fact that electron‐withdrawing groups can reduce the electron cloud density of sulfonyl hydrazone N─H bonds, enhance the hydrogen bond donor activity of molecules, and form more stable hydrogen bond interactions with enzyme active residues. While electron‐donating substituted compounds (**5b** and **5c**) showed slightly lower enzymatic inhibitory activity, their antioxidant free radical scavenging ability was relatively improved, which is due to the fact that electron‐donating groups can provide electrons to neutralize free radicals and optimize antioxidant performance. In addition, the poly‐substituted benzene ring derivative 5g showed decreased comprehensive activity, indicating that excessive steric hindrance of aromatic ring substituents will offset the electronic optimization effect and reduce molecular bioactivity, which explains the abnormal activity difference of individual compounds in the experiment.

Most importantly, the dual pharmacophore synergistic effect is the core source of the excellent performance of the target compounds. The rigid thiochroman‐4‐one skeleton provides stable hydrophobic anchoring effect for the enzyme active pocket, while the flexible sulfonyl hydrazone fragment provides abundant polar interaction sites. The combination of hydrophobic effect, hydrogen bond interaction and electrostatic force constructs a multi‐dimensional stable binding system, which makes the hybrid derivatives significantly superior to single‐skeleton thiochroman‐4‐one or sulfonyl hydrazone compounds reported in literatures in terms of target affinity and pharmacological activity.

### 3.4 Molecular Simulation Mechanism In‐Depth Discussion

Molecular docking and 100 ns MD simulation were used to clarify the atomic‐level binding mechanism between the optimal compound **5k** and α‐glucosidase. Docking results showed that compound **5k** could stably bind to the enzyme active pocket through multiple interactions. MD simulation trajectory analysis further verified the stability of the ligand‐protein complex. The RMSD, *R*_g_ and buried SASA values of the compound **5k**‐α‐glucosidase complex tended to be stable throughout the simulation process, indicating that the ligand would not dissociate from the active pocket and the overall structure of the complex was compact and stable. The RMSF results showed that the amino acid residues near the ligand binding site had low flexibility, confirming that the binding of compound **5k** could effectively stabilize the spatial conformation of α‐glucosidase and inhibit its catalytic activity. Hydrogen bond analysis showed that compound **5k** could form high‐frequency stable hydrogen bonds with PHE995 and other key residues, and the continuous hydrogen bond interaction was the key to maintaining the long‐term stable binding of the complex. MMPBSA energy decomposition analysis further proved that van der Waals interaction dominated the binding process, supplemented by electrostatic and hydrophobic interactions, and the total binding free energy was −49.76 kJ/mol, indicating excellent target affinity of compound **5k**.

### 3.5 Drug‐Likeness and ADMET Potential Evaluation

Drug‐likeness and ADMET prediction are essential indicators to evaluate the druggable potential of lead compounds and guide subsequent drug development. In this study, all synthesized derivatives fully complied with Lipinski's rule of five, and the optimal compound **5k** had ideal molecular weight, lipophilicity and hydrogen bond donor–acceptor characteristics, laying a good foundation for oral bioavailability. ADMET prediction results further confirmed that compound **5k** had favorable absorption, distribution, metabolism and excretion properties, as well as good biological safety, no obvious toxic and side‐effect risks, which meets the basic screening criteria of candidate small‐molecule drugs.

### 3.6 Research Limitations and Future Prospects

Although this study systematically completed the design, synthesis, bioactivity evaluation, and mechanism simulation of novel thiochroman‐4‐one derivatives, there are still some limitations. First, the current pharmacological evaluation is limited to in vitro level, and in vivo hypoglycemic, antioxidant, and pharmacodynamic verification of optimal compounds need to be supplemented. Second, the enzyme kinetic inhibition mechanism and cellular‐level antidiabetic mechanism need further in‐depth exploration. In addition, the structural optimization of target compounds can be further carried out based on the summarized SAR rules, such as screening more high‐quality substituents to balance dual activity and further improve druggability. In future work, we will carry out in vivo animal experiments, cellular mechanism research and structural modification of lead compounds, and further promote the preclinical research and application of such novel multi‐functional small‐molecule drugs.

## 4 Conclusions

In conclusion, a total of 11 novel thiochroman‐4‐one derivatives incorporating a sulfonyl hydrazone moiety were prepared. Bioassay results indicated that compound **5k** showed remarkable α‐glucosidase inhibitory activity (IC_50_ = 4.57 µg/mL), approximately 51‐fold more potent than acarbose. Meanwhile, compound **5j** exhibited excellent antibacterial activity against *Xac* (EC_50_ = 15.67 µg/mL), superior to commercial bactericides. In addition, molecular simulations revealed that compound **5k** binds stably to α‐glucosidase mainly through van der Waals interactions. ADMET and drug‐likeness analyses validated the druggable potential of compound **5k**. These findings support further optimization of thiochroman‐4‐one–sulfonyl hydrazone hybrids as promising antidiabetic and antibacterial candidates.

## 5 Experimental Section

### 5.1 General

The uncorrected melting points (mp) were determined using a WRX‐4 binocular microscope (Shanghai Yice Technology Instrument Co., Ltd., Shanghai, China). ^1^H NMR and ^13^C NMR spectra were recorded on a Bruker Advance DRX‐400 NMR spectrometer (Bruker, Rheinstetten, Germany). High‐resolution mass spectrometry (HRMS) data were acquired using a Waters Xevo G2‐XS QTOF mass spectrometer (Waters, Milford, MA, USA). All reagents employed in this study were of analytical or chemical grade and purchased from the Chinese Chemical Reagent Company.

### 5.2 General Procedure for the Synthesis of the Target Compounds

As shown in Scheme 1, in a 250 mL round‐bottom flask, a solution containing 4‐substituted thiophenols (**1**, 100 mM), chloropropionic acid (120 mM, 1.2 equivalents), and NaOH (150 mM, 1.5 equivalents) in deionized water (100 mL) was heated at 50°C for 3–5 h. Upon completion, the mixture was acidified to a pH range of 1–2 by adding 5% aqueous HCl (50 mL). The resulting solid product was collected by filtration and purified via recrystallization from ethanol to afford pure intermediate **2**. Subsequently, intermediate **2** (80 mM) was dissolved in pre‐chilled concentrated H_2_SO_4_ (100 mL) in a 250 mL round‐bottom flask and stirred under an ice bath for 2–4 h. After reaction completion, the mixture was poured into ice water (100 mL), and the precipitate formed was isolated by filtration and recrystallized from methanol to yield intermediate **3**. For the preparation of intermediate **4**, intermediate **3** (50 mM), acetic acid (50 mL), and 30% hydrogen peroxide (150 mM, 3 equivalents) were placed in a 100 mL three‐necked flask. The reaction was carried out at 50°C for 3–5 h, then quenched by pouring into deionized water (50 mL). The solid that formed was purified by recrystallization from absolute ethanol to obtain intermediate **4**. Intermediate **4** (10 mmol), substituted benzenesulfonyl hydrazide (12 mmol, 1.2 equivalents), absolute ethanol (25 mL), and acetic acid (1 mL) were introduced into a 50 mL round‐bottomed flask. The reaction mixture was then heated under reflux for 2–4 h. After the reaction was completed, the crude products were separated by filtration and purified through recrystallization from ethanol to obtain the target compounds **5a**−**5k**.

#### 5.2.1 (*E*)‐*N*'‐(6‐Methyl‐1,1‐Dioxidothiochroman‐4‐Ylidene)Benzenesulfonohydrazide (**5a**)

White solid, mp 214.4°C–216.1°C, yield 67%; ^1^H NMR (400 MHz, DMSO‐d_6_, ppm) *δ*: 11.09 (s, 1H, NH), 8.09 (dd, *J* = 8.0, 4.0 Hz, 2H, Ar─H), 7.74–7.64 (m, 5H, Ar─H), 7.45 (dd, *J* = 8.0, 4.0 Hz, 1H, Ar─H), 3.66 (t, *J* = 8.0 Hz, 2H, CH_2_), 3.16 (t, *J* = 8.0 Hz, 2H, CH_2_), 2.39 (s, 3H, CH_3_); ^13^C NMR (100 MHz, DMSO‐d_6_, ppm) *δ*: 146.36, 143.85, 139.12, 135.50, 133.84, 132.54, 131.54, 129.68, 128.13, 126.25, 123.26, 46.25, 25.20, 21.73; HRMS (ESI) [M+Na]^+^ calcd for C_16_H_16_N_2_O_4_S_2_: 387.044370, found 387.04378.

#### 5.2.2 (*E*)‐4‐Methyl‐*N*'‐(6‐Methyl‐1,1‐Dioxidothiochroman‐4‐Ylidene)Benzenesulfonohydrazide (**5b**)

White solid, mp 229.9°C–231.1°C, yield 69%; ^1^H NMR (400 MHz, DMSO‐d_6_, ppm) *δ*: 10.97 (s, 1H, NH), 7.86 (d, *J* = 8.0 Hz, 2H, Ar─H), 7.71 (t, *J* = 8.0 Hz, 2H, Ar─H), 7.45 (dd, *J* = 8.0, 4.0 Hz, 3H, Ar─H), 3.64 (t, *J* = 8.0 Hz, 2H, CH_2_), 3.12 (t, *J* = 8.0 Hz, 2H, CH_2_); 2.41 (s, 3H, CH_3_), 2.38 (s, 3H, CH_3_); ^13^C NMR (100 MHz, DMSO‐d_6_, ppm) *δ*: 146.03, 144.33, 143.84, 136.23, 135.48, 132.59, 131.49, 130.10, 128.16, 126.22, 123.25, 46.21, 25.15, 21.76, 21.52; HRMS (ESI) [M+Na]^+^ calcd for C_17_H_18_N_2_O_4_S_2_: 401.060020, found 401.05844.

#### 5.2.3 (*E*)‐4‐Methoxy‐*N*'‐(6‐Methyl‐1,1‐Dioxidothiochroman‐4‐Ylidene)Benzenesulfonohydrazide (**5c**)

White solid, mp 231.0°C–232.8°C, yield 71%; ^1^H NMR (400 MHz, DMSO‐d_6_, ppm) *δ*: 10.91 (s, 1H, NH), 7.94–7.90 (m, 2H, Ar─H), 7.72 (t, *J* = 8.0 Hz, 2H, Ar─H), 7.45 (dd, *J* = 8.0, 4.0 Hz, 1H, Ar─H), 7.14 (dd, *J* = 8.0, 4.0 Hz, 2H, Ar─H), 3.83 (s, 3H, OCH_3_), 3.65 (t, *J* = 8.0 Hz, 2H, CH_2_), 3.14 (t, *J* = 8.0 Hz, 2H, CH_2_), 2.41 (s, 3H, CH_3_); ^13^C NMR (100 MHz, DMSO‐d_6_, ppm) *δ*: 163.37, 145.90, 143.85, 135.45, 132.62, 131.45, 130.63, 130.38, 126.25, 123.24, 114.82, 56.16, 46.24, 25.14, 21.75; HRMS (ESI) [M+Na]^+^ calcd for C_17_H_18_N_2_O_5_S_2_: 417.054934, found 417.05464.

#### 5.2.4 (*E*)‐*N*'‐(6‐Fluoro‐1,1‐Dioxidothiochroman‐4‐Ylidene)Benzenesulfonohydrazide (**5d**)

Yellow solid, mp 224.8°C–226.1°C, yield 64%; ^1^H NMR (400 MHz, DMSO‐d_6_, ppm) *δ*: 11.30 (s, 1H, NH), 8.00–7.92 (m, 3H, Ar─H), 7.73–7.64 (m, 3H, Ar─H), 7.56‐7.48 (m, 2H, Ar─H), 3.73 (t, *J* = 8.0 Hz, 2H, CH_2_), 3.19 (t, *J* = 8.0 Hz, 2H, CH_2_); ^13^C NMR (100 MHz, DMSO‐d_6_, ppm) *δ*: 164.61 (d, *J* = 249.0 Hz), 145.11 (d, *J* = 2.0 Hz), 139.01, 135.86 (d, *J* = 9.0 Hz), 134.63 (d, *J* = 3.0 Hz), 133.94, 129.79, 128.07, 126.81 (d, *J* = 10.0 Hz), 118.41 (d, *J* = 23.0 Hz), 112.24 (d, *J* = 25.0 Hz), 46.08, 25.29; HRMS (ESI) [M+Na]^+^ calcd for C_15_H_13_FN_2_O_4_S_2_: 391.019298, found 391.01931.

#### 5.2.5 (*E*)‐*N*'‐(6‐Fluoro‐1,1‐Dioxidothiochroman‐4‐Ylidene)‐4‐Methylbenzenesulfonohydrazide (**5e**)

White solid, mp 197.8°C–199.1°C, yield 60%; ^1^H NMR (400 MHz, DMSO‐d_6_, ppm) *δ*: 11.19 (s, 1H, NH), 7.92 (dd, *J* = 8.0, 4.0 Hz, 1H, Ar─H), 7.85 (d, *J* = 8.0 Hz, 2H, Ar─H), 7.56–7.44 (m, 4H, Ar─H), 3.71 (t, *J* = 8.0 Hz, 2H, CH_2_), 3.13 (t, *J* = 8.0 Hz, 2H, CH_2_), 2.38 (s, 3H, CH_3_); ^13^C NMR (100 MHz, DMSO‐d_6_, ppm) *δ*: 164.45 (d, *J* = 249.0 Hz), 144.81, 144.45, 136.12, 135.92 (d, *J* = 9.0 Hz), 134.60 (d, *J* = 3.0 Hz), 130.21, 128.10, 126.82 (d, *J* = 9.0 Hz), 118.37 (d, *J* = 23.0 Hz), 112.19 (d, *J* = 24.0 Hz), 46.05, 25.24, 21.51; HRMS (ESI) [M+Na]^+^ calcd for C_16_H_15_FN_2_O_4_S_2_: 405.034948, found 405.03349.

#### 5.2.6 (*E*)‐*N*'‐(6‐Fluoro‐1,1‐Dioxidothiochroman‐4‐Ylidene)‐4‐Methoxybenzenesulfonohydrazide (**5f**)

White solid, mp 175.4°C–177.1°C, yield 68%; 1H NMR (400 MHz, DMSO‐d_6_, ppm) *δ*: 11.16 (s, 1H, NH), 7.97–7.94 (m, 3H, Ar─H), 7.61 (dd, *J* = 8.0, 4.0 Hz, 1H, Ar─H), 7.53–7.48 (m, 1H, Ar─H), 7.20–7.16 (m, 2H, Ar─H), 3.84 (s, 3H, OCH_3_), 3.74 (t, *J* = 8.0 Hz, 2H, CH_2_), 3.22 (t, *J* = 8.0 Hz, 2H, CH_2_); ^13^C NMR (100 MHz, DMSO‐d_6_, ppm) *δ*: 164.64 (d, *J* = 249.0 Hz), 163.43, 144.66 (d, *J* = 2.0 Hz), 135.96 (d, *J* = 9.0 Hz), 134.55 (d, *J* = 3.0 Hz), 130.51, 130.33, 126.77 (d, *J* = 10.0 Hz), 118.39 (d, *J* = 23.0 Hz), 114.91, 112.26 (d, *J* = 25.0 Hz), 56.13, 46.11, 25.25; HRMS (ESI) [M+Na]^+^ calcd for C_16_H_15_FN_2_O_5_S_2_: 421.029862, found 421.02940.

#### 5.2.7 (*E*)‐*N*'‐(6‐Fluoro‐1,1‐Dioxidothiochroman‐4‐Ylidene)‐2,4,6‐Trimethylbenzenesulfonohydrazide (**5g**)

White solid, mp 193.2°C–194.4°C, yield 67%; ^1^H NMR (400 MHz, DMSO‐d_6_, ppm) *δ*: 11.46 (s, 1H, NH), 7.92 (dd, *J* = 8.0, 4.0 Hz, 1H, Ar─H), 7.50–7.45 (m, 1H, Ar─H), 7.36 (dd, *J* = 8.0, 4.0 Hz, 1H, Ar─H), 7.10 (s, 2H, Ar─H), 3.72 (t, *J* = 8.0 Hz, 2H, CH_2_), 3.15 (t, *J* = 8.0 Hz, 2H, CH_2_), 2.68 (s, 6H, 2 × CH_3_), 2.26 (s, 3H, CH_3_); ^13^C NMR (100 MHz, DMSO‐d_6_, ppm) *δ*: 164.53 (d, *J* = 249.0 Hz), 143.35, 142.38, 140.00, 136.22 (d, *J* = 8.0 Hz), 134.46, 133.26, 132.21, 126.86 (d, *J* = 10.0 Hz), 118.05 (d, *J* = 23.0 Hz), 111.84 (d, *J* = 25.0 Hz), 46.03, 25.18, 23.17, 20.91; HRMS (ESI) [M+Na]^+^ calcd for C_18_H_19_FN_2_O_4_S_2_: 433.066248, found 433.06601.

#### 5.2.8 (*E*)‐*N*'‐(6‐Chloro‐1,1‐Dioxidothiochroman‐4‐Ylidene)Benzenesulfonohydrazide (**5h**)

White solid, mp 237.1°C–239.0°C, yield 74%; ^1^H NMR (400 MHz, DMSO‐d_6_, ppm) *δ*: 11.32 (s, 1H, NH), 7.96 (d, *J* = 8.0 Hz, 2H, Ar─H), 7.86 (d, *J* = 8.0 Hz, 1H, Ar─H), 7.77–7.64 (m, 5H, Ar─H), 3.73 (t, *J* = 8.0 Hz, 2H, CH_2_), 3.15 (t, *J* = 8.0 Hz, 2H, CH_2_); ^13^C NMR (100 MHz, DMSO‐d_6_, ppm) *δ*: 144.49, 138.40, 136.69, 136.16, 134.70, 130.71, 130.20, 128.04, 125.61, 125.39, 46.00, 25.18, 21.50; HRMS (ESI) [M+Na]^+^ calcd for C_15_H_13_ClN_2_O_4_S_2_: 406.989747, found 406.98928.

#### 5.2.9 (*E*)‐*N*'‐(6‐Chloro‐1,1‐Dioxidothiochroman‐4‐Ylidene)‐4‐Methylbenzenesulfonohydrazide (**5i**)

Yellow solid, mp 222.9°C–224.4°C, yield 70%; ^1^H NMR (400 MHz, DMSO‐d_6_, ppm) *δ*: 11.24 (s, 1H, NH), 7.87–7.80 (m, 4H, Ar─H), 7.71 (dd, *J* = 8.0, 4.0 Hz, 1H, Ar─H), 7.45 (d, *J* = 8.0 Hz, 2H, Ar─H), 3.73 (t, *J* = 8.0 Hz, 2H, CH_2_), 3.16 (t, *J* = 8.0 Hz, 2H, CH_2_), 2.38 (s, 3H, CH_3_); ^13^C NMR (100 MHz, DMSO‐d_6_, ppm) *δ*: 144.54, 144.49, 138.40, 136.69, 136.16, 134.70, 130.71, 130.20, 128.04, 125.61, 125.39, 46.00, 25.18, 21.50; HRMS (ESI) [M+Na]^+^ calcd for C_16_H_15_ClN_2_O_4_S_2_: 421.005397, found 421.00472.

#### 5.2.10 (*E*)‐*N*'‐(6‐Chloro‐1,1‐Dioxidothiochroman‐4‐Ylidene)‐4‐Methoxybenzenesulfonohydrazide (**5j**)

Yellow solid, mp 193.2°C–194.4°C, yield 67%; ^1^H NMR (400 MHz, DMSO‐d_6_, ppm) *δ*: 11.15 (s, 1H, NH), 7.89 (d, *J* = 8.0 Hz, 2H, Ar─H), 7.86 (d, *J* = 8.0 Hz, 1H, Ar─H), 7.80 (d, *J* = 4.0 Hz, 1H, Ar─H), 7.71 (dd, *J* = 8.0, 4.0 Hz, 1H, Ar─H), 7.17 (d, *J* = 8.0 Hz, 2H, Ar─H), 3.84 (s, 3H, OCH_3_), 3.73 (t, *J* = 8.0 Hz, 2H, CH_2_), 3.14 (t, *J* = 8.0 Hz, 2H, CH_2_); ^13^C NMR (100 MHz, DMSO‐d_6_, ppm) *δ*: 163.44, 144.37, 138.40, 136.67, 134.74, 130.68, 130.52, 130.26, 125.61, 125.40, 56.21, 45.99, 25.16; HRMS (ESI) [M+Na]^+^ calcd for C_16_H_15_ClN_2_O_5_S_2_: 437.000312, found 436.99904.

#### 5.2.11 (*E*)‐*N*'‐(6‐Chloro‐1,1‐Dioxidothiochroman‐4‐Ylidene)‐2,4,6‐Trimethylbenzenesulfonohydrazide (**5k**)

Yellow solid, mp 229.4°C–230.6°C, yield 62%; ^1^H NMR (400 MHz, DMSO‐d_6_, ppm) *δ*: 11.46 (s, 1H, NH), 7.92 (dd, *J* = 8.0, 4.0 Hz, 1H, Ar─H), 7.50–7.45 (m, 1H, Ar─H), 7.36 (dd, *J* = 8.0, 4.0 Hz, 1H, Ar─H), 7.10 (s, 2H, Ar─H), 3.72 (t, *J* = 8.0 Hz, 2H, CH_2_), 3.15 (t, *J* = 8.0 Hz, 2H, CH_2_), 2.68 (s, 6H, 2 × CH_3_), 2.26 (s, 3H, CH_3_); ^13^C NMR (100 MHz, DMSO‐d_6_, ppm) *δ*: 143.37, 142.05, 140.01, 138.27, 136.53, 134.99, 133.29, 132.21, 130.33, 125.60, 125.32, 45.98, 25.07, 23.17, 20.92; HRMS (ESI) [M+Na]^+^ calcd for C_18_H_19_ClN_2_O_4_S_2_: 449.036697, found 449.03643.

### 5.3 Antioxidant and Anti‐α‐Glucosidase Assays

The in vitro antioxidant (as demonstrated by their DPPH and ABTS radical scavenging capabilities), anti‐α‐glucosidase, and antibacterial activities of the synthesized compounds **5a–5k** was evaluated following the protocol described in our previous methods [13, 43].

#### 5.3.1 DPPH Radical Scavenging Assay

To generate the stock solution, 5 mg of each test compound was accurately weighed and dissolved in 100 mL of methanol, resulting in a primary stock concentration of 50 µg/mL. For the DPPH assay, 160 µL of this stock solution was combined with 40 µL of freshly prepared 0.5 mM DPPH solution in a 96‐well microplate, giving a final assay concentration of 40 µg/mL. The mixture was then shielded from light and allowed to react at room temperature for 30 min. Absorbance readings at 517 nm were acquired using a Multiskan Sky microplate spectrophotometer (Thermo Fisher Scientific, China). As controls, dimethyl sulfoxide (DMSO) served as the blank (negative control), and ascorbic acid (vitamin C) was employed as the positive reference standard to benchmark the antioxidant activity of the tested compounds.

#### 5.3.2 ABTS Radical Scavenging Assay

A fresh ABTS working solution was prepared immediately before each assay. To generate the ABTS stock solution, equal volumes of 2.6 mM potassium persulfate (K_2_S_2_O_8_) and 7.4 mM ABTS were mixed and allowed to react for 12 h in the dark at ambient temperature. This pre‐incubated stock was then diluted 50‐fold using phosphate‐buffered saline (PBS, pH 7.4) to yield the working solution used in subsequent measurements. Each test compound was precisely weighed and dissolved in methanol, followed by dilution to a final volume of 100 mL to obtain a stock concentration of 62.5 µg/mL. For the assay, 160 µL of this stock solution was mixed with 40 µL of the diluted ABTS working solution in a 96‐well plate, resulting in a final sample concentration of 50 µg/mL. Following a 30‐min incubation in darkness, absorbance was measured at 734 nm using a Multiskan Sky microplate spectrophotometer (Thermo Fisher Scientific, Shanghai, China). DMSO was employed as the blank control, while trolox was used as the standard antioxidant reference.

#### 5.3.3 Α‐Glucosidase Inhibitory Activity Assay

All test compounds were initially solubilized in 100 µL of DMSO, followed by dilution with 900 µL of phosphate‐buffered saline (PBS, pH 6.8) to yield a primary stock solution at 4000 µg/mL for the α‐glucosidase inhibition assay. For each well of a 96‐well microplate, the reaction mixture was assembled in the following order: 20 µL of α‐glucosidase (adjusted to a final activity of 0.5 U/mL), 70 µL of PBS, 80 µL of 0.2 M sodium carbonate (Na_2_CO_3_), and 10 µL of the appropriately diluted test compound solution. The plate was then incubated at 37°C for 10 min to allow equilibration of enzyme–substrate conditions. Subsequently, 20 µL of p‐nitrophenyl‐α‐D‐glucopyranoside (PNPG) was added to achieve a final substrate concentration of 2.5 mM; under these conditions, the test compound's final concentration in the assay was 200 µg/mL. The enzymatic reaction proceeded for an additional 30 min at 37°C. Reaction termination was achieved by measuring the absorbance of the liberated p‐nitrophenol at 405 nm using a Multiskan Sky microplate spectrophotometer (Thermo Fisher Scientific, Shanghai, China), enabling quantitative assessment of α‐glucosidase inhibition.

### 5.4 Molecular Simulation

Molecular simulation is a key in silico technique widely employed to investigate how biologically active molecules bind to and interact with large biomolecular targets [44]. Human intestinal α‐glucosidase (PDB ID: 3TOP) plays a central role in carbohydrate metabolism by hydrolyzing complex dietary polysaccharides into absorbable glucose units, thereby influencing both digestive efficiency and postprandial glycemic control [45]. To elucidate the binding mode and key interaction forces between the potent derivative **5k** and this enzyme, we performed molecular docking simulations using AutoDock Tools 1.5.6, following our previously established protocol [22]. MD simulation (100 ns) was conducted using GROMACS 2022.2 with the Amber14SB and GAFF2 force fields. Binding free energy was calculated by the MM‐PBSA method [46, 47, 48, 49].

### 5.5 ADMET and Drug‐Likeness Analysis

In this investigation, the ADMET (absorption, distribution, metabolism, excretion, and toxicity) profile and drug‐like properties of compound **5k** were assessed on December 29, 2025, employing the Deep‐PK platform (https://biosig.lab.uq.edu.au/deeppk/) and the SwissADME tool (https://www.swissadme.ch/), respectively [50, 51].

### 5.6 Antibacterial Activity Assay

In addition to the core antidiabetic pharmacological evaluation (α‐glucosidase inhibition and antioxidant activity), this study additionally evaluated the auxiliary antibacterial activity of the target compounds against *Xac* using the turbidimetric method [15].

Each target compound (7.5 mg) was first solubilized in 150 µL of DMSO. Aliquots of 80 and 40 µL from this stock solution were then separately dispensed into two 15 mL centrifuge tubes, each pre‐filled with 4 mL of an aqueous 0.1% Tween 80 solution. From these diluted mixtures, 1 mL was withdrawn and transferred to individual test tubes containing 4 mL of nutrient broth (NB), yielding final test concentrations of 200 and 100 µg/mL, respectively. Subsequently, 40 µL of NB medium, pre‐inoculated with *Xac* at an optical density (OD_595_) of 0.6–0.8, was added to each tube. The cultures were incubated at 30°C under agitation (180 rpm) for 24–48 h, or until the OD_595_ reached the target range of 0.6–0.8. The inhibition rates were determined using the established protocol described in reference [15].

This antibacterial activity is an independent and complementary pharmacological property, which is not involved in the antidiabetic molecular mechanism, nor incorporated into the hypoglycemic structure–activity relationship. The evaluation aims to explore the multi‐functional application potential of the novel thiochroman‐4‐one derivatives, and the relevant activity data are only used for the comprehensive pharmacological property analysis of the compounds, without affecting the core antidiabetic research conclusion of this study.

### 5.7 Data Analysis

The in vitro scavenging activities against DPPH and ABTS free radicals, as well as the inhibitory effect on α‐glucosidase, were calculated and expressed as mean values ± standard deviation (SD) using the specified formula. In parallel, IC_50_ or EC_50_ values, representing the concentration required for 50% inhibition, were derived through GraphPad Prism 5 software (San Diego, CA, USA). All biological experiments were performed with three biological replicates to ensure data reproducibility.

$$\mathrm{Inhibition}\;\mathrm{rate}\;(\%)=\frac{{\mathrm{OD}}_{\mathrm{control}}-{\mathrm{OD}}_{\mathrm{sample}}}{{\mathrm{OD}}_{\mathrm{control}}}\times 100\%$$

## Author Contributions

**Pei Li**: funding acquisition and writing – review and editing. **Wenmin Pan**: investigation and methodology. **Tiantian Zhuang**: investigation, formal analysis, and writing – original draft. **Xiang Wang**: supervision, funding acquisition, and writing – review and editing.

## Conflicts of Interest

The authors declare no conflicts of interest.

## Supporting information




Supporting Information for this article is available on the WWW under https://doi.org/10.1002/cbdv.71884.
**Supporting File 1**: cbdv71884‐sup‐0001‐SuppMat.docx.

## Data Availability

The data that support the findings of this study are available from the corresponding author upon reasonable request.
