# Supplementary material for: Novel Thiochroman‐4‐One Derivatives Bearing a Sulfonyl Hydrazone Moiety: Synthesis, Bioactivity, Molecular Simulation, and Drug‐Likeness Evaluation
Source: Chem Biodivers. 2026 Oct 27;23(10):e71884. doi: 10.1002/cbdv.71884 (PMC13711463; doi:10.1002/cbdv.71884)
Supplement: Supplementary file 1 [file CBDV-23-e71884-s001.docx]

**Table S1**. Predicted ADMET parameters of compound **5k**.

| ADMET parameters | ADMET name | Physicochemical properties |
| --- | --- | --- |
| Absorption | Human oral bioavailability 20% | Bioavailable |
|  | Human intestinal absorption | Absorbed |
|  | Human oral bioavailability 50% | Bioavailable |
|  | p-Glycoprotein inhibitor | Inhibitor |
|  | p-Glycoprotein substrate | Non-substrate |
|  | Skin permeability | Low skin permeability: log KP > -2.5 |
| Distribution | Blood-brain barrier | Non-penetrable |
| Metabolism | CYP 1A2 inhibitor | Non-inhibitor |
|  | CYP 1A2 substrate | Substrate |
|  | CYP 2C19 inhibitor | Inhibitor |
|  | CYP 2C19 substrate | Substrate |
|  | CYP 2C9 inhibitor | Non-inhibitor |
|  | CYP 2C9 substrate | Substrate |
|  | CYP 2D6 inhibitor | Non-inhibitor |
|  | CYP 2D6 substrate | Non-substrate |
|  | CYP 3A4 inhibitor | Inhibitor |
|  | CYP 3A4 substrate | Substrate |
|  | OATP1B1 | Non-inhibitor |
|  | OATP1B3 | Non-inhibitor |
| Excretion | Organic cation transporter 2 | Non-inhibitor |
|  | Half-life of drug | < 3hs |
| Toxicity | AMES Mutagenesis | Safe |
|  | Avian | Safe |
|  | Biodegradation | Safe |
|  | Carcinogenesis | Safe |
|  | Crustacean | Safe |
|  | Eye corrosion | Safe |
|  | Eye irritation | Safe |
|  | hERG blockers | Safe |
|  | NR-AhR | Safe |
|  | NR-AR | Safe |
|  | NR-AR-LBD | Safe |
|  | NR-aromatase | Safe |
|  | NR-ER | Safe |
|  | NR-ER-LBD | Safe |
|  | NR-GR | Safe |
|  | NR-PPAR-gamma | Safe |
|  | NR-TR | Safe |
|  | Respiratory disease | Safe |
|  | Skin sensitisation | Safe |
|  | SR-ATAD5 | Safe |
|  | SR-HSE | Safe |
|  | SR-MMP | Safe |
|  | SR-p53 | Safe |

**Table S2**. Drug-likeness parameters of compound **5k**.

| Drug-likeness parameters | Physicochemical properties |
| --- | --- |
| Molecular mass (MW) | 426.94 |
| logP | 3.49 |
| Hydrogen bond donors | 1 |
| Hydrogen bond acceptors | 5 |

^1^H NMR spectra of compound **5a**

^13^C NMR spectra of compound **5a**

HRMS spectra of compound **5a**

^1^H NMR spectra of compound **5b**

^13^C NMR spectra of compound **5b**

HRMS spectra of compound **5b**

^1^H NMR spectra of compound **5c**

^13^C NMR spectra of compound **5c**

HRMS spectra of compound **5c**

^1^H NMR spectra of compound **5d**

^13^C NMR spectra of compound **5d**

HRMS spectra of compound **5d**

^1^H NMR spectra of compound **5e**

^13^C NMR spectra of compound **5e**

HRMS spectra of compound **5e**

^1^H NMR spectra of compound **5f**

^13^C NMR spectra of compound **5f**

HRMS spectra of compound **5f**

^1^H NMR spectra of compound **5g**

^13^C NMR spectra of compound **5g**

HRMS spectra of compound **5g**

^1^H NMR spectra of compound **5h**

^13^C NMR spectra of compound **5h**

HRMS spectra of compound **5h**

^1^H NMR spectra of compound **5i**

^13^C NMR spectra of compound **5i**

HRMS spectra of compound **5i**

^1^H NMR spectra of compound **5j**

^13^C NMR spectra of compound **5j**

HRMS spectra of compound **5j**

^1^H NMR spectra of compound **5k**

^13^C NMR spectra of compound **5k**

HRMS spectra of compound **5k**
